# Metamaterials-Enabled Sensing for Human-Machine Interfacing

**DOI:** 10.3390/s21010161

**Published:** 2020-12-29

**Authors:** Fei Li, Run Hu

**Affiliations:** 1 School of Art, Hubei University, Wuhan 430062, China; lifei@hubu.edu.cn; 2 School of Energy and Power Engineering, Huazhong University of Science and Technology, Wuhan 430074, China

**Keywords:** metamaterials, human-machine interface, sensor

## Abstract

Our modern lives have been radically revolutionized by mechanical or electric machines that redefine and recreate the way we work, communicate, entertain, and travel. Whether being perceived or not, human-machine interfacing (HMI) technologies have been extensively employed in our daily lives, and only when the machines can sense the ambient through various signals, they can respond to human commands for finishing desired tasks. Metamaterials have offered a great platform to develop the sensing materials and devices from different disciplines with very high accuracy, thus enabling the great potential for HMI applications. For this regard, significant progresses have been achieved in the recent decade, but haven’t been reviewed systematically yet. In the Review, we introduce the working principle, state-of-the-art sensing metamaterials, and the corresponding enabled HMI applications. For practical HMI applications, four kinds of signals are usually used, i.e., light, heat, sound, and force, and therefore the progresses in these four aspects are discussed in particular. Finally, the future directions for the metamaterials-based HMI applications are outlined and discussed.

## 1. Introduction

Ever since humans invented machines, people have begun to operate them, and in broad sense, the human-machine interaction, no matter being noticed or not, also began. The purpose of inventing mechanical machines was originally to assist humans in finishing some repeated, burdensome or dangerous processing task, and later on the concept of machine became broader to include electronic devices, which have doubtlessly changed our daily lives revolutionarily [1,2,3]. Nowadays, no one will deny that electronic machines and devices have played such an important role that they have changed, redefined, recreated the way we work, sense, communicate, entertain, travel, and interact with the world. For example, nowadays we work on computers, we sense the temperature and humidity by sensors, we communicate with each other by cellphones and emails, we entertain on virtual-reality (VR)/augmented-reality (AR)/mixed-reality (MR) devices, we read the global news on computers/cellphones/radios, we drive vehicles/airplanes/ships, etc. [4,5,6,7,8]. According to the application and functionality scenarios, electronic machines can be sorted into, including but not limited to, manufacturing machinery, living appliances, working appliances, medical devices, entertainment equipment, etc. From the interactive approach, the HMI can be sorted into mechanical (touch, press, stretch) [3,9], acoustic (sound) [10,11], optics (light) [12,13], thermal (infrared or bionic) [14,15,16], and so on. Along with the advent of machines, people have began to interact with these machines, but the human-machine interface (HMI) concept was firstly proposed and systematically investigated in the 1980s [2]. Since then, the HMI has been attracting more sustained attention and research interest in more sensitive, flexible, and reliable interactions with machines from the perspective of materials, devices, systems, and algorithms [17,18,19,20,21,22,23,24]. For practical and feasible HMI, accurate sensing of the signals from human beings is the first step. Only when the machines sense the human commands using signals like light, heat, sound, or force, can they respond to the commands to achieve the desired tasks. Therefore, accurate and multi-discipline sensing is extremely demanded for HMI research and application.

Metamaterials, as a kind of artificial materials whose basic structural units are much smaller than the resonant wavelength, show extraordinary electromagnetic (EM) properties beyond the conventional nature materials [25,26,27]. When EM waves are incident onto metamaterials, the electric and magnetic fields can be coupled to induce the so-called size effect against viewing the microscopic details of the metamaterial structures by resolving the electric or magnetic waves. Inhomogeneous microscopic structures can be regarded as a continuous substance whose effective electric permittivity and magnetic permeability can be modified to be positive, zero, and negative at certain wavelength to exhibit some abnormal phenomena, such as negative refraction [28,29,30,31], diffraction-limit breaking imaging [32], super-resolution focusing/cloaking [33,34,35], super-lens [36,37,38,39], etc. Although metamaterials were first proposed to manipulate EM waves, the concept of metamaterials has been extended to cover the full wavelengths of EM and other disciplines, like thermotics [34,40,41,42,43,44,45,46], acoustics [11,47], magnetics [37], electrics [48,49], matter wave [50], and mechanics [51]. 

Since the effective parameters of metamaterials can be tuned by the structures, the maximum effective parameters correspond to a strong localization and enhancement of the underlying fields. Such local maxima in the corresponding spectra or signals of metamaterials can be used as the eigenvalues or indications to improve the sensing accuracy and selectivity to enable the super-resolution detection of ambient environment, even with extremely small amounts of analytes [52,53]. For example, most EM metamaterials involve metallic nanostructures because metal nanostructures enable the occurrence of the localized surface plasmon resonance (LSPR) phenomenon, which is the collective but non-propagating oscillations of surface electrons in the metal nanostructures [54,55,56,57,58,59,60,61,62]. LSPR can lead to the significant EM enhancement, altering the light-matter interaction with different absorption, reflection, and transmission properties, resulting in different characterization techniques like plasmon-enhanced fluorescence, surface-enhanced infrared absorption spectroscopy, and surface-enhanced Raman scattering [63]. By detecting the small shifts in frequency of resonance, we can differentiate the refractive index of the local environment for sensing applications. 

When attaching a metamaterial-based strain sensor onto an object, the corresponding resonance peak will be shifted upon the external mechanical properties, thus the resonance signal can also be used for mechanical or acoustic sensing. More specific, acoustic metamaterials can be designed to possess very-high positive or negative mass densities, bulk moduli, and refractive index, thus offering unprecedented opportunities for acoustic sensing via the enhanced directivity and sensitivity. Due to this extraordinary sensing capability, metamaterials have been used for sensing, catalysis, spectroscopy, imaging, etc. There have existed a lot of sensing metamaterials, such as nanodisks [64,65], nanorods [12,66,67,68], nanoshells [69], nanoantennas [70], nanorings [71], etc. The metamaterials are size scalable, enabling a wide range of sensing applications including optical sensing, infrared sensing, microwave sensing, acoustic sensing, and mechanical sensing. 

**Figure 1:**
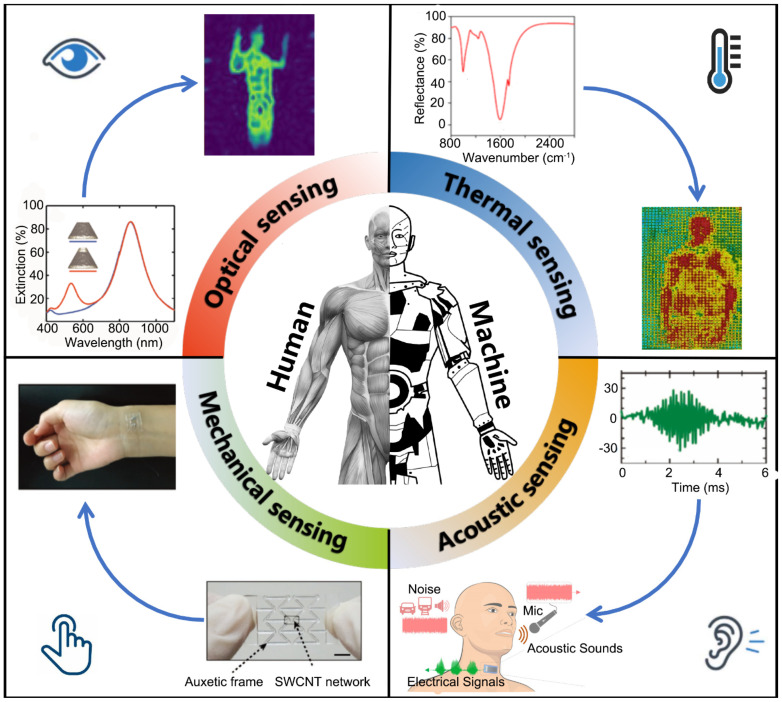
Metamaterial-enabled sensing for human-machine interfacing, including optical sensing, thermal sensing, acoustic sensing, and mechanical sensing, respectively. In each quadrant, the sensing signals and typical HMI applications are illustrated.

Note that the accurate sensing is extremely demanded for HMI and metamaterials have exhibited such great potential for sensing applications, it is naturally to combine these two together and develop the metamaterials-based sensing materials and devices for HMI applications. It is, of course, unsurprised that a lot of effort has been devoted to exploring this field and significant progresses have been achieved in the past decades. As far as we are concerned, these progresses have not been reviewed and introduced systematically and comprehensively yet. In this Review, we would like to focus on the introduction of the metamaterials-enabled sensing for HMI applications, ranging from the working principle, the materials synthesis, the device development, to the application scenarios. For practical HMI applications, four kinds of signals are usually used, i.e., light, heat, sound, and force, as shown in Figure 1, and therefore the progresses in these four aspects are discussed in particular. Finally, the future research directions and promising applications are outlined and discussed. 

## 2. Metamaterials-Based Sensing Principles

When LSPR happens between the metamaterials and the incident waves happens, the resonance frequency can be calculated as [63]:

(1) $$\omega_{\mathrm{LSPR}}=\frac{\omega_{p}}{\sqrt{1+2\varepsilon_{d}}}=\sqrt{\frac{ne^{2}}{m_{eff}\varepsilon_{0}(1+2\varepsilon_{d})}}$$

where *ω_p_* is called as the plasma frequency, which is the characteristic frequency of the oscillation electrons in the metal nanostructures and can be calculated as $\omega_{p}=\sqrt{n_{d}e^{2}/m_{eff}\varepsilon_{0}}$. *n_d_* and *e* are the density of electrons and charge of an electron. *m_eff_* is the effective mass of electrons, and *ε*_0_ and *ε_d_* are and the permittivity of free space and the dielectric surrounding medium, respectively. The plasma frequency is such an important concept that corresponds to how easily the electrons can be stimulated by the incident EM waves, and how EM waves behaves upon the interface. When an EM wave is incident onto the metallic metamaterials with a frequency higher than the plasma frequency, the electrons near the metal surface will not oscillate and the EM wave will be simply transmitted or absorbed by the intraband transition. Otherwise, electron oscillation will occur and the EM wave will be reflected instead. The environment-dependent resonance frequency in Equation (1) will be shifted and thereby EM field will be enhanced with several orders of magnitude stronger than the incident field strength. This enables the accurate sensing of the changes of the local environment. 

Besides plasmonic metamaterials, the Mie-resonance metamaterials also exhibit electric or magnetic resonance, paving another way for sensing metamaterials. From Mie theory, the electric and magnetic dipoles contribute significantly to the effective permittivity (*ε_eff_*) and effective permeability (*μ_eff_*) of the particle composite as [72]:

(2) $$\frac{\varepsilon_{eff}}{\varepsilon_{1}}=\frac{\frac{F(\theta )+2b_{e}}{F(\theta )-b_{e}}+2\nu_{f}}{\frac{F(\theta )+2b_{e}}{F(\theta )-b_{e}}-\nu_{f}},\;\frac{\mu_{eff}}{\mu_{1}}=\frac{\frac{F(\theta )+2b_{m}}{F(\theta )-b_{m}}+2\nu_{f}}{\frac{F(\theta )+2b_{m}}{F(\theta )-b_{m}}-\nu_{f}}$$

where $F(\theta )=\frac{2(\sin\theta -\theta \cos\theta )}{(\theta^{2}-1)\sin\theta +\theta \cos\theta }$, *b_e_* and *b_m_* are the permittivity and permeability ratio of the background matrix to the magnetodielectric spheres, and *ν_f_* is the volume fraction. As *F*(*θ*) is a resonant function, negative permittivity or permeability can be achieved corresponding to the large electric or magnetic fields in the so-called Mie metamaterials. More than spherical particles, the cubic, cylindrical, and ellipsoid particles are also demonstrated to achieve Mie resonance. By utilizing these Mie resonance spectra, metamaterial-enabled sensing can also be achieved. 

To denote the sensing capability, the refractive index sensitivity can be denoted by:

(3) $$S=\frac{\Delta \lambda_{p}}{\Delta n}\;\mathrm{nm}/\mathrm{RIU}$$

with the refractive index unit (RIU) where *λ_p_* is the plasma wavelength and *n* is the refractive index. Usually, the wavelength and energy sensitivities of the plasmonic metamaterials are in the several 100~1000 nm/RIU and several 10~100 meV/RIU, respectively [14,73]. Some researchers prefer to use the figure-of-merit (FOM = *S*/FWHM) for evaluating the sensing performance of metamaterials, where FWHM is the full width at half maximum of the resonance peaks [74,75,76]. In broad concept, the optical, infrared, microwave metamaterials are all EM metamaterials and share the similar working principle of sensing by detecting the small frequency shift of the resonance spectra.

When converting the acoustic and mechanical signals into resonance spectra, the metamaterials can also be applied for acoustic and mechanical sensing according to Equation (1). Moreover, the specific acoustic pressure field, can also be used for acoustic sensing by analyzing the spatial oscillation and spectral response along the propagation direction through the metamaterials, which can be explicitly described as [11,47]:

(4) $$P(z,f)=\frac{\sqrt{2\pi \rho_{air}f}\sqrt[{4}]{1-n_{eff}^{-2}}}{\cos[\arctan(\frac{\rho_{x}}{\rho_{air}}\sqrt{n_{eff}^{2}-1})]}\exp\left[ik_{air}\int_{z_{o}}^{z}n_{eff}dz\right]$$

where *n_air_* is the refractive index of air, *f* is the frequency of the acoustic wave, *z* is the distance, *ρ* is the effective mass density. A strong wave compression effect can be observed in acoustic metamaterials provides a hint for acoustic sensing. For mechanical sensing, the sensitivity can be defined as the gauge factor by converting the mechanical stimuli into electrical signals [77]:

(5) $$\mathrm{GF}(\xi )=\frac{\Delta R(\xi )}{R_{0}}\frac{1}{\xi }\;$$

where ∆*R*(*ξ*)/*R*_0_ is the relative resistance change and *ξ* is the tensile strain. As long as we can integrate different kinds of electrical resistive sensors or electrical capacitive sensors onto the machine skin, this is the most practical way for HMI. For this regard, these have existed various ways like thin films, fibers and textiles, electronic skins, and so on. 

## 3. Metamaterials-Enabled Sensing

### 3.1. Optical Sensing

**Figure 2:**
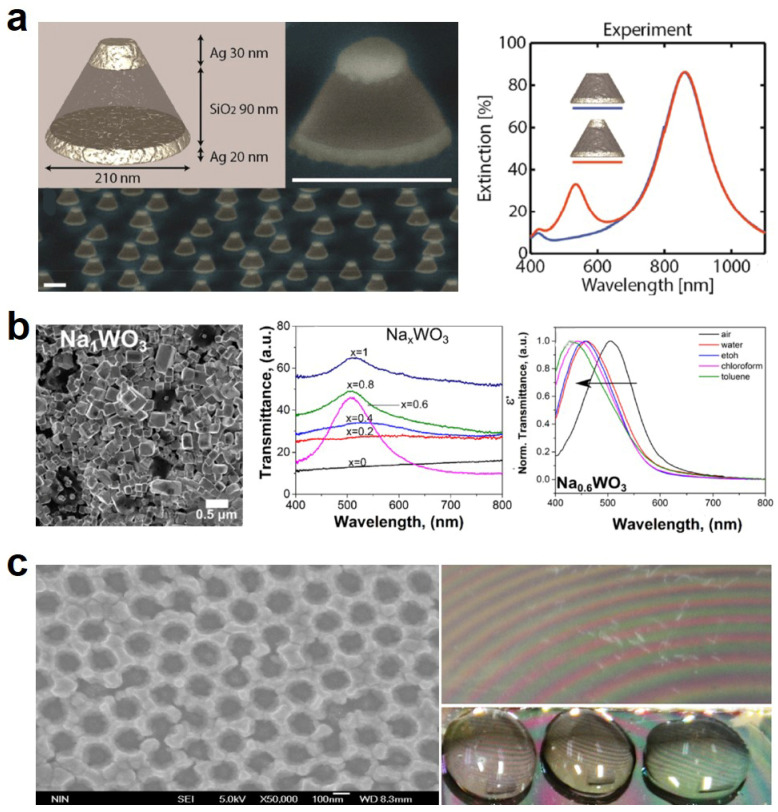
Optical metamaterial for sensing. (**a**) Schematic and SEM images of the 3D metasurface sensor, experimental optical extinction spectra w/o the top disk. Reproduced with permission from [64]. Copyright 2020, American Chemical Society. (**b**) SEM image of Na_x_WO_3_-based ENZ metamaterials UV-vis transmittance and normalized transmittance of Na0.6WO3 metamaterials at different environments and wavelengths. Reproduced with permission from [73]. Copyright 2018, American Chemical Society. (**c**) SEM image of the porous silver film, and the digital photographs of the sensor, and with water droplet, 15% glucose solution droplet, and 60% sucrose solution droplet. Reproduced with permission from [84]. Copyright 2013, American Institute of Physics.

As mentioned above, metamaterials stem from the control and amplificariong of the light-matter interaction effect, based on which the well-designed structured surface can response to the change of surrounding in terms of optical properties like the refractive index. Usually, most metamaterials are the two-dimensional array of nanoplasmonic structures on solid surfaces, based on which many sensing devices are developed. However, it is perceived that the 3D metamaterials will show more sensitive and accurate sensing properties due to the enhanced stereoscopic detecting areas and spatial detecting processes at the nanoscale in 3D. Nugroho et al. fabricated a quasi-random array of truncated silver/SiO2/silver nanocone-based structures, as shown in Figure 2a, to enable simultaneous and independent optical sensing [64]. The material combinations were quantified to shift the LSPR peaks within the visible and near-infrared (NIR) ranges. The corresponding optical extinction spectra (red curve) show that one peak was at 860 nm and the other was at 535 nm. When the top silver disk was moved, the visible sensing LSPR peak disappeared, which implies that the 3D metamaterial structure play an important role for sensing application. To further avoid silver oxidation and enhance the temperature stability, they further coated Si_3_N4 disk on the top the silver disk by plasma-enhanced chemical vapor deposition technique, but without shifting the LSPR peaks. The bulk refractive index sensitivity is 133 nm/RIU for the NIR peak, and 180 nm/RIU for the visible peak. In contrast, the local or thin film sensitivity is denoted by the decay length, which is 49 nm for the visible peak and 27 nm for the NIR peak. The 3D sensing functionality was finally characterized by mearing the glass transition temperature as the 3D metamaterials were embedded in different polymers. 

To avoid the high loss at the visible band due to the high imaginary part of the permittivity of some metamaterials, the epsilon-near-zero (ENZ) metamaterials, whose real part of permittivity may approach zero, show much intrinsic superiority over conventional materials. ENZ metamaterials emerge in recent years with a lot of functionalities and applications, such as invisible cloaking, nano-optical circuits, nonlinear optics and so on, but the practical fabrication remains challenging [78,79,80,81,82,83]. Fusco et al. engineered a kind of nanostructured sodium tungsten bronze (Na_x_WO_3_) ENZ metamaterials for achieving optical sensing [73]. As shown in Figure 2b, the SEM image of NaWO_3_ shows the formation of cubic nanocrystals after high-temperature reduction. The UV-vis transmittance curves show that when the Na/W ratio reached 0.6, the transmittance was highest with largest quality factor, enabling the optical sensing application. Further, by exposing the ENZ metamaterials at different environment, the transmittance peaks blue shifted by about 100 nm. The optical sensitivity was 150 nm/RIU, which was competing against other sensing devices. One drawback of most metamaterials-based optical sensors is that the optical sensing depends on the characterization of the refractive index through the spectrophotometers or refractometers. More ideal way should be through our naked eyes. Wang et al. fabricated a two-layer structure of an orderly arranged porous silver film and a silver film on polyvinyl alcohol (PVA) with distinct refractive indexes [84]. The reflected light in such a two-layer structure interfered with red to green fringes in air. When dropping different liquid droplets onto the metamaterials, the different fringes can be detected by naked eyes for identifying the different liquids, as shown in Figure 2c. Such direct optical sensing enables a convenient and useful way for HMI applications. 

### 3.2. Thermal Sensing

**Figure 3:**
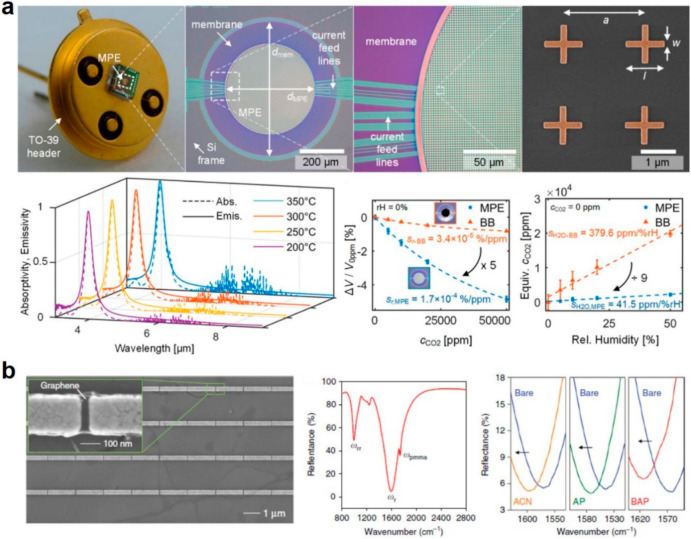
Thermal metamaterials for sensing. (**a**) Hierarchical structure of the infrared sensing device, experimental absorptivity and emissivity spectra at different temperatures, sensitivity characterization at various CO_2_ concentration and humidity. Reproduced with permission from [91]. Copyright 2017, American Society of Chemistry. (**b**) SEM image of graphene-coated nanorod antennas, the reflectance spectrum, and the reflectance spectra before and after chemical functionalization. Reproduced with permission from [92]. Copyright 2017, American Society of Chemistry.

Thermal/infrared sensing is more promising in HMI applications since it can be used to sense objects and environments through the infrared light as all objects emit infrared light at anytime and anywhere [14,15,16,85,86,87,88,89,90]. Lochbaum et al. combined microelectromechanical system (MEMS) and metamaterial perfect emitter to achieve narrowband on-chip infrared emitter for infrared sensing [91]. The hierarchical structure of the infrared sensing device is shown in Figure 3a, which consists of a complementary metal oxide semiconductor (CMOS) substrate, a MEMS hotplate, a metamaterial perfect emitter (MPE) and an aluminum oxide (Al_2_O_3_) sealing layer from bottom to top. The MEMS hotplate can be heated up to 450 ℃, and the MPE is used to tune the thermal radiation by the wavelength-selective emissivity engineering. At the resonance frequency, the metal-insulator-metal (MIM) structure of the MPE induces a strong but confined magnetic field at the top resonators, which enables the emissivity engineering for thermal/infrared sensing. The good agreement of the measured absorptivity and the emissivity at different temperatures validates the Kirchhoff’s law. The normalized coefficient of the resonance bandwidth of the absorptivity and emissivity are 5.8 × 10^−4^/℃ and 5.4 × 10^−4^/℃ respectively. To demonstrate the gas sensing application, they fabricated a system where different gases were blown onto the metamaterial detector. The comparison of the normalized voltage difference ∆V/V0ppm between the presented MPE and a blackbody (BB) emitter demonstrated that the relative sensitivity increases from 3.4 × 10^−5^ to 1.7 × 10^−4^%/ppm due to the larger factional absorptivity performance of the MPE. While the cross-sensitivity of the MPE to humidity was also demonstrated to be as minimal as 41.5 ppm/% rH, which is 9-fold smaller than that of the BB emitter. Two-dimensional materials, such as graphene, are also very good candidates for optical sensing or photodetector in the infrared range. Zhu et al. demonstrated a metasurface-based infrared sensing through a monolayer graphene and a golden nanorod array [92]. As shown in Figure 3b, graphene was suspended on the golden antenna array with 30 nm gaps. The golden nanorods were fabricated on a Pt mirror with a SiO_2_ spacer layer in between, where the latter two layers formed the optical cavity for plasmonic resonance. Due to such resonance, two resonance dips in the reflectance spectrum were observed at 1000 and 1500 cm^−1^ in the infrared band, where the latter one was used as the primary plasmonic resonance band for infrared sensing with stronger absorption. When exposing to different environment including acetonitrile (ACN), aminopyrene (AP) in ACN, and boronic acid-pyrene (BAP) in ACN, the reflectance spectrum shifted by 23, 27, and 46 cm^−1^, respectively. It is because of the enhancement of graphene conductivity that the reflectance spectrum blue shifted. Such blue shifts can be used for high-sensitivity biosensing for small molecules. 

### 3.3. Acoustic Sensing

Acoustic metamaterials (AMMs), as a kind of artificial periodic structure to manipulate sound waves with smaller-than-wavelength unit cells, have been proposed and developed rapidly with many emerging applications [93,94,95,96,97]. Among them, acoustic sensing is developed for acoustic imaging, detection, navigation, communication, health monitoring, and medical therapy [95,98]. For HMI application, it is rather important to enhance the detection, localization, and communication capabilities with high directivity and high sensitivity. However, to achieve these is not an easy task especially under the complicated conditions with noise, multi-speaker, weak-intensity interference. A conventional way is resorting to phased-array technology with microphone array systems, whose bulky sizes limits the integration with HMI. As alternatives, piezoresistive, piezoelectric, capacitive, and optical-based sensors have been reported to achieve acoustic sensing, but usually, their detectable pressure is rather small [99,100]. Along with way toward high-performance acoustic sensing, AMMs offer unprecedented opportunities to advance acoustic sensing by their extraordinary properties like negative-equivalent or gradient density, bulk modulus, and refractive index through modulating the dispersion of materials and structures [10]. As typical examples, various AMMs are reported to enhance sound emission and reception, like phononic crystals [101], graded refractive index (GRIN) AMMs [102], Mie resonant AMMs [13], anisotropic AMMs [10], near-zero-index (NZI) AMM [11], etc. 

**Figure 4:**
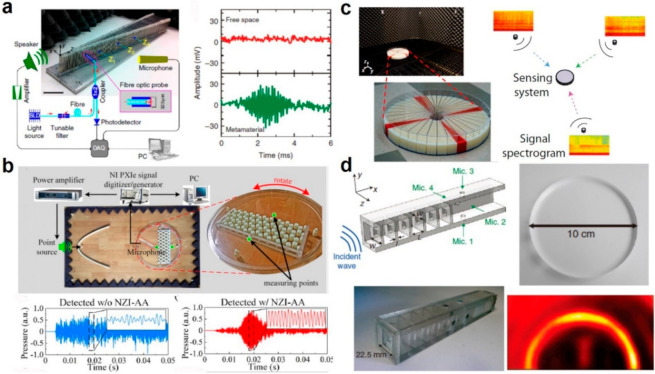
Acoustic metamaterials for sensing. (**a**) Schematic of the AMM hybrid sensing system, time-domain pulse signal in free space and in the metamaterial. Reproduced with permission from [96]. Copyright 2014, Nature. (**b**) Experimental setup for characterizing the NZI-AA sample on a rotating table, and time-domain pressure with and without the NZI-AA sample. Reproduced with permission from [11]. Copyright 2019, American Institute of Physics. (**c**) Fabricated metamaterial sensor and the schematic for the single-sensor listening device for sound detection from several sources. Reproduced with permission from [95]. Copyright 2015, PNA. (**d**) Schematic of the AMM with five symmetrical resonators, 3D printed prototype, optical image and the detected edge of a 10-cm-diameter disc. Reproduced with permission from [97]. Copyright 2015, Nature.

Chen et al. developed a GRIN AMM-enhanced acoustic sensing system to allow sound propagation in high-refractive-index medium with enhanced pressure field [96]. GRIN AMMs offer possibilities to slow down and even trap acoustic waves, and the phenomenon has a bright name as rainbow trapping. The key component for the AMM sensing system was a linearly tapered metadevice with GRIN distribution along the sound propagation direction. Thanks to GRIN metamaterials, the effective refractive index increases, and the wavelength gradually decreases, results in a spatial compression of the acoustic wave and a remarkable directional response. With GRIN metamaterials, there is a strong wave compression effect along propagation direction, which enables the advanced acoustic sensing with enhanced sound intensity sensing and sound source localization. The fabricated AMM and the experimental setup are shown in Figure 4a. Based on such setup, the time-domain acoustic pulse signals in the free space and within GRIN metamaterials are measured. It is seen that the free-space signal is overwhelmed by the noise with signal-to-noise ratio (SNR) at 0.27 while detected through the present AMM. It is demonstrated that such AMM-based acoustic sensing system overcome the detection limit of a conventional acoustic sensor (SNR at 1). Ma et al. fabricated an NZI AMM sensing system for combined highly directive-sensitive detection [11]. NZI AMMs offer near-zero relative mass density and refractive index, which benefits the sound tunneling to overcome the detection limit of current acoustic technologies. They selected a phononic crystal with a honeycomb lattice of solid cylinders in air. With special modulation of the lattice constant and periodic pitch, the configuration forms a special band structure with a double Dirac cone which can be regarded as NZI medium. To fabricate such sensing system, they manufactured the NZI device with epoxy resin via 3D printing and assembled onto a turntable with precise angle increment of 1°, as shown in Figure 4b. Very high directivity and weak signal amplification were demonstrated. To detect signals from strong interference noise, the time-domain signals without and with the NZI AMM are measured. It is seen that the transmitted sinusoidal pulse signal is strongly disturbed by the noise, while is resolved again by the present AMM sensing system. 

Other than pursuing high directivity and high sensitivity for acoustic sensing, AMM can also be used to solve the multi-speaker listening problem which is more practical in application scenarios. Xie et al. designed a multi-speaker listening system by modulating an array of Helmholtz resonators to tune the frequency dispersion [95]. As shown in Figure 4c, the system is composed by a single sensor surrounded by 36 fan-shape AMM waveguides. The measurement modes of the single sensor at different frequencies are different, which can be utilized to solve the multi-speaker listening problem via spatiotemporal encoding and decoding processing. They further developed an inverse algorithm to segregate the mixed sound signal and reconstruct the audio content of each sound source. Such flexible sensing capability is desired to achieve multisource speech recognition and segregation for HMI applications. Besides, AMM also can be used to enhance machine vision with edge detection. Moleron and Daraio proposed an AMM that consists of an array of coupled resonators-based waveguiding structure, as shown in Figure 4d [97]. The dimensions of the structure were specifically designed to generate the frequency-dependent coincidence of the tapered resonances and the plane mode bandgap, which induce a spectral band to allow the transmission of waves with large perpendicular wavenumber. To demonstrate the subwavelength edge detection performance, they imaged a 10 cm-diameter Plexiglass disc and observed clear edges. By visualizing the intensity components, they also observed the horizontal and vertical edges separately. The unique subwavelength edge detection stems from the combination of tapered resonance and Bragg scattering that enables and disables the propagation of evanescent waves, respectively. 

### 3.4. Mechanical Sensing

**Figure 5:**
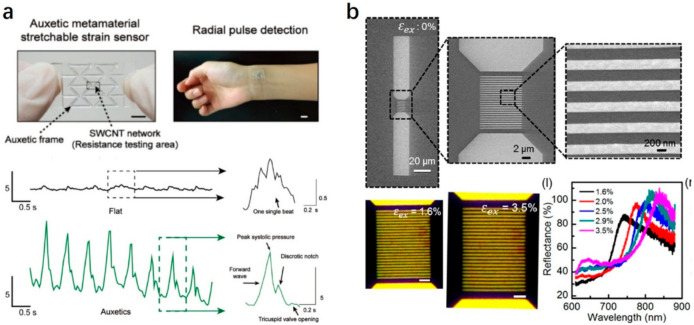
Mechanical metamaterials for sensing. (**a**) Photograph of the auxetic metamaterial stretchable mechanical sensor for radial pulse detection, the detected human pulse profiles by a conventional flat sensor and the metamaterial sensor. Reproduced with permission from [106]. Copyright 2018, Wiley. (**b**) SEM images of the mechanical metasurface on a PDMS substrate without external strain, optical microscope images of the metasurface under 1.6% and 3.5% strain, experimental reflectance of the metasurface with various strains. Reproduced with permission from [77]. Copyright 2018, American Chemical Society.

Compared to previous sensing functionalities, the mechanical sensing is more important for HMI applications as touching, pressing, stretching, and twisting are our basic ways to interact with surrounding objects. The reason why we put the mechanical sensing at last is because metamaterials were born for EM applications initially and gradually applied to other disciplines including magnetics, thermotics, acoustics, mechanics, and so on [27]. Mechanical sensing is usually achieved by converting the mechanical stimuli into readable electrical or optical signals, and is well applied in wearable electronics, electronic skins, smart textiles, healthcare monitoring, soft robotics, etc. [103,104]. The conventional materials, when stretching, usually expand in one direction while compress in the orthogonal direction due to the conservation of mass and volume. This is our common sense and easy to understand. But due to such behavior, the stretchable sensitivity is intrinsically limited as the distance between two materials increases obeying the transverse Poisson compression under longitudinal stretching. Good news comes that mechanical metamaterials, in contrast to traditional materials, can exhibit extraordinary mechanical behaviors like negative structured Poisson’s ratio, mechanical instability, compressible tunability, etc. [51,100,103,104,105]. Thus, mechanical metamaterials can be used to overcome such limitations for mechanical sensing. Jiang et al. developed an auxetic mechanical metamaterial sensor, which is in contrast to traditional materials that can expand in both directions under stretching [106]. Thus, the mechanical sensitivity is greatly enhanced. They integrated the single-wall carbon nanotube (SWCNT) network onto polydimethylsiloxane (PDMS) thin film with a PDMS auxetic frame, as shown in Figure 5a. Upon stretching, on the one hand, the frame could tune the Poisson’s ratio to expand in both directions, and on the other hand, the microcracks propagated in SWCNT network and changed the electrical resistance. The GF was increased to 835, which is almost 24-fold improvement over traditional sensors. For HMI applications, they further mounted the metamaterials sensor into human wrist to monitor the radial pulse wave with high SNR of 104.8 dB. The difference of pulse peaks of the metamaterials sensor and a traditional sensor was obvious; thus, the metamaterials sensor could be applied for HMI for continuous daily health monitoring. Previous example is to convert the mechanical stimuli into electrical signals, and it also can be converted into optical signals. Chen et al. fabricated golden plasmonic metasurface on PDMS surface to amplify the mechanical sensitivity [77]. The SEM images of the metasurface with increasing magnification at zero external strain (ε_ex_ = 0%) are shown in Figure 5b. When light is incident onto the metasurfacce, it is polarized along the surface and its diffractive orders will superposed to generate the Rayleigh anomalies. Under different external strains from 1.6% to 3.5%, the LSPR peaks will shifted linearly, which can be used as the indicator of the external strain with high mechanical sensitivity at about 48 nm/1% external strain, far beyond the state-of-the-art stretchable plasmonic resonators. 

## 4. Enabled HMI Applications

In a broad concept, metamaterials are a kind of artificial materials and exhibit exotic properties over conventional naturally-existing materials. Due to the enhanced resonance performance, metamaterials have shown much potential for accurate sensing, and have been utilized for HMI applications with some significant progress. Here, we also summarize these progresses from the aspects of optical, thermal, acoustic, and mechanical sensing. 

### 4.1. HMI Based on Optical/Microwave Sensing 

**Figure 6:**
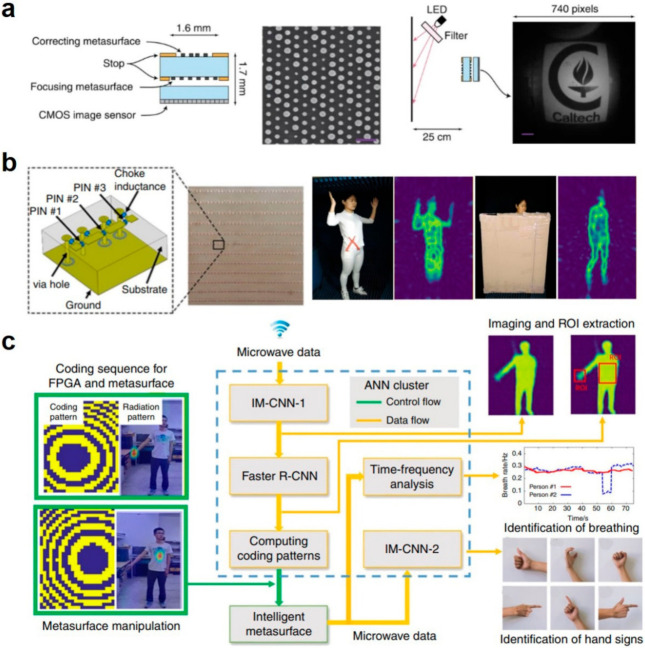
Optical metamaterials for human detection. (**a**) Schematic for the miniature planar metasurface-based camera, SEM image of the top view of the metasurface, the imaging setup and the experimental image obtained by the metasurface camera. Reproduced with permission from [108]. Copyright 2016, Nature. (**b**) Schematic and photograph of the coding metasurface and the unit cell, photographs of the human body and the reconstructed images by the metasurface-based machine-learning imager directly or through a plate. Reproduced with permission from [112]. Copyright 2019, Nature. (**c**) Microwave data processing flow by deep learning CNN cluster for respiration monitoring and sign-language recognition. Reproduced with permission from [114]. Copyright 2019, Nature.

In our daily life, optical sensing is the most widely used way for us to sense the world through eyes. As such, most existing HMI applications also employ cameras to sense the world. Camera have been used to “see” the ambient and machines then do some preset responses to be “smart”, like the unmanned automobiles and robots. Especially with the advent of VR/AR/MR technologies, the virtual environment that created by the special cameras have offered great promise as alternative ways for HMI though the head-mounted displays, flat projection surfaces, and fully enclosed environment, etc. [20,107]. Conventional cameras are usually composed by assembling discrete bulk lens and filters, and precise alignment is required to achieve acceptable imaging performance. Such bulky-size optical systems limit HMI application, partially at least, as these head-mounted display and glasses require low-weight and accurate sensing devices. In such cases, optical metasurfaces and metamaterials offer lots of merits to overcome such limitations as they can be fabricated in planar configuration and aligned lithographically to avoid the complicated post-fabrication alignment [108]. Such planar optical lens comes from the so-called perfect lens, which is one of the most ground-breaking applications of optical metamaterials with negative refractive index [38,39,109,110,111]. Some metamaterial lenses have been applied to focus or collimate light beams from an optical fiber or a semiconductor laser, but they still suffer from monochromatic aberrations and limited field of view. Arbabi et al. demonstrated a doublet lens, whose top is the correcting metasurface and whose bottom is a focusing metasurface, to overcome such sufferings efficiently, as shown in Figure 6a [108]. Based on the doublet metamaterial lens, they further integrated with a CMOS image sensor to enable an ultrathin, planar, low f-number metamaterial camera. The metasurface is made by hexagonal array of amorphous silicon nano-posts with varying diameters on the top of fused silica, as shown by the SEM image in the middle of Figure 6a. With the home-made experimental setup, where the pattern was placed 25 cm away from the camera and illuminated by an LED, they obtained the image with a wide field of view in Figure 6a. The imaging performance could be further enhanced by using a monochromatic image sensor with a smaller pixel size. By post evaluation, the metamaterial camera is found to process merits like high efficiency and high incident angle tolerance. 

Beyond optical cameras, the microwave cameras are also exhibit the great potential for HMI applications although microwaves are not visual by our eyes but can be detected by the microwave sensors, here the metamaterial sensor for instance. Both optical cameras and microwave cameras share the similar working principle as EM wave, and the only difference is the working frequency range. Conventional microwave cameras require rapid data processing and large data storage capability, disabling the real-time and in-situ reconstruction of desired scenes and images intelligently and efficiently [10,112,113]. As a result, the imaging speed and quality are usually compromised in most existing microwave cameras. Li et al. tackled this challenge by combining machine learning techniques with 2-bit metasurfaces, and demonstrated the feasibility to produce high image quality and high accuracy without heavy image reconstructions [112]. The 2-bit coding metasurface is constructed by a 2D array of meta-atoms and subwavelength-scale patches on a dielectric substrate. A PIN diode was used to connect the patches and denote the “00”, “01”, “10”, and “11” states controlled by the applied bias voltage, corresponding to the four digitized scattering phase levels at 0, π/2, π, 3π/2, respectively. After training, they applied the metamaterial camera to monitor a moving person. The optical images and the corresponding reconstructed images in Figure 6b demonstrate that not only the gestures of the person but also the “see-through-the-wall” ability can be achieved by the machine-learning metamaterial cameras. The same group, as shown in Figure 6c, further developed an intelligent metasurface convolutional neural network (IM-CNN) to reconstruct the image of a human body, to monitor the respiration from the region of interest (ROI) of the whole image, to recognize the hand gesture with additional CNN or the time-frequency analysis of the microwave data [114]. The metasurface-based CNN not only can exclude unwanted disturbances but also can enhance the SNR by a factor of 20 dB. The present metasurface system not only can “see” what people are doing but also can “hear” what people are talking even against a wall or complex background noises, offering great potential for intelligent HMI applications. 

### 4.2. HMI Based on Thermal Sensing

**Figure 7:**
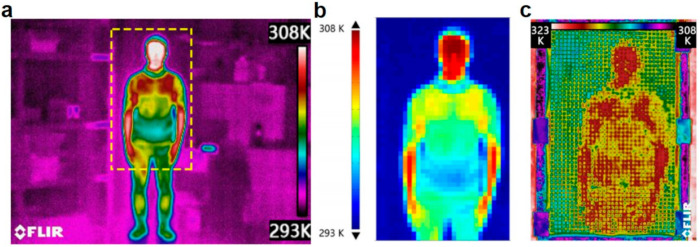
Thermal metamaterials for human recognition. (**a**) infrared image of a human body. (**b**) Digital discretization of the original infrared image to 31 × 51 = 1581 pixels. (**c**) experimental infrared image of the digital metasurface to show the thermograms of a human body. Reproduced with permission from [116]. Copyright 2018, American Institute of Physics.

Compared to optical signals, thermal/infrared signals are emitted and received almost wherever and whenever as any objects above 0 K will emit thermal radiation, thus more and more HMI scenarios refer to thermal sensing. For example, an increasing number of modern weapons are equipped with thermal signal detectors, like thermal imagers or infrared cameras, to enhance the target localization capability especially at nighttime. When equipped with thermal and infrared sensors, machines or robots can also detect the ambient temperature or heat flux, and then achieve preset or dynamic responses. Erden and Cetin reported a multimodal hand gesture recognition system based on infrared sensors [115]. Hand movement was first detected by the sensor array, and then examined by a video camera. More than gesture recognition, Shang et al. designed a digital thermal metasurface to generate the infrared thermograms of the human body [116]. They extracted an infrared image with 31 × 51 = 1581 pixels, which correspond to 1581 different thermal conductivities. Then they fabricated an acrylic plate with 1581 holes; each hole stands for a pixel. Such digital discretization will of course generate information entropy loss compared to the original infrared image. It is perceived that the more pixels the plate has, the clearer the infrared images will be. After the space discretization step, they used the thermal conductivity discretization step with only ten kinds of thermal conductivity units to fill the plate, and the experimental infrared image was shown in Figure 7. Regardless of the rough interfaces, we can see the human image as designed. Such thermal metasurface can be utilized to dynamically generate the thermograms for potential HMI applications. 

### 4.3. HMI Based on Acoustic Sensing

**Figure 8:**
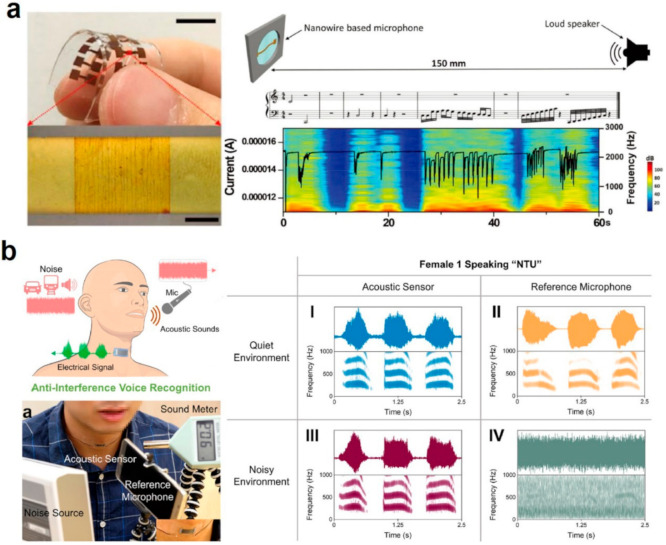
Acoustic metamaterials for sound recognition. (**a**) Photograph of the soft nanowire-based acoustic metamaterial membrane, schematic of the experimental setup of music detection, and acoustic sensor output (curve) and the STFT analysis of the background in response to notes with different music scale. Reproduced with permission from [117]. Copyright 2020, Wiley. (**b**) Schematic and photograph of the flexible anti-interference voice recognition acoustic metamaterial sensor, performance comparison of the phonation of “NTU” by the fabricated acoustic sensor and a reference microphone at quiet and noisy environments. Reproduced with permission from [119]. Copyright 2020, American Society of Chemistry.

As smart devices are becoming smaller and smaller in size, the conventional mechanical touching or pressing on the rigid or flexible screen may result in mistouches or errors, leading to the inconvenience for HMI applications. At such a scenario, wireless gesture recognition is in high demand. For HMI, the sensitive detecting of the incoming sound is the first step. Such acoustic sensors can play an important role not only in HMI but also in some healthcare and medical applications. Hearing impairment has become a global issue, suffered by nearly 10% of the world population. Cochlear implants can convert sound signals into electrical stimuli for the auditory nerves, but most of the cochlear implants are rigid, thus sometimes cause wearing discomfort and nerve injury. Gong et al. designed a suspended local-crack nanowire metamaterial acoustic sensor to detect the out-of-plane sound signals with rather-good static and dynamic acoustic frequency discrimination capability up to 3000 Hz [117]. The acoustic metamaterial sensor prototype is shown in Figure 8a, which is soft and durable. They further measure the electrical output from the acoustic sensor, the resistance changes of the acoustic sensor agreed with the spectrograms of the music sound from a 150-mm-away loudspeaker. The working frequency ranges from 319 to 1951 Hz with high acoustic sensitivity of 0.48–4.26 Pa^−1^, within the human communication frequency range, which enables the HMI applications including voice-based control of prosthetics, drones, and robot. 

Compared to detecting the sound frequency, it is more important to recognize the human voice for HMI applications. As such, the human voice can be directly used as commands to control and manipulate the machines or robots. To recognize human voices, one challenge remains that the voice must be clearly detected against different background noises and situations [10,11,47,95,96,97]. To achieve this, the acoustic sensor is required to have high sensitivity, low detection limit, fast response behavior, high mechanical flexibility and reliability, and it is more desired to have water repellency to enhance the application adaptability against skin moisture and air moisture. Though extensive efforts have been devoted to developing acoustic sensors with different strategies, materials, and structures, but the satisfactory performances (sensitivity, SNR, response time, etc.) are still under exploring [47,100,118]. Le et al. fabricated a graphene-based ultra-sensitive acoustic sensor for anti-interference voice recognition [119]. The multiscale microcracks in the conductive thin film can respond to the ambient acoustic signals, resulting in the ultrahigh sensitivity (GF = 8699), ultralow detection limit, ultrafast response time, and excellent durability over 10,000 cycles. Figure 8b shows the flexible acoustic sensor on a speaker’s neck, and the comparison of the phonation of “NTU” recorded by the acoustic sensor and a reference microphone at quiet and noisy environments respectively. The comparisons clearly demonstrate that the fabricated sensor could discriminate the phonation patterns at both quiet and noisy environments, showing great superiority over the reference microphone and the ultrasensitive anti-interference voice recognition. They further tested a sentence voice signal and very good voice recognition was also demonstrated. These demonstrations clearly exhibit the potential HMI application based on acoustic metamaterial sensing.

### 4.4. HMI Based on Mechanical Sensing

Compared to other sensing signals, the mechanical signals may be the most widely used for HMI applications as mechanical sensing is a human-like way to interact with the ambient. We are used to sense objects by touching and pressing the surface of the objects to distinguish the materials, structure, and shape, and then to hold, lift, pull, and push objects for desired purposes. Most of the existing machines or robots aim at taking this human-like capability as the first step to demonstrate the capability to finish some given tasks. Therefore, the mechanical sensing is more basic for HMI applications and most research interests for advanced robots or HMI demonstrations are focused on the materials and devices improvement in terms of the mechanical sensing performance. Previously, people have used different functional materials to sense or emulate a wide range of mechanical stimuli such as pressure and strain, like piezoelectric materials, triboelectric materials, etc. [2,120,121,122,123,124,125,126,127,128]. However, the soft stretching capability is one of remaining issues to be overcome that is rather preferred for practical HMI applications as rigid materials are incompatible to the skins, uncomfortable to wear, and lacking of adaptability to the dynamic motion of human body [128,129]. For this regard, the electronic skins and smart textiles show more superiority for next-generation mechanical sensing for HMI, and have been extensively explored in terms of the performances, fabrications, and applications [2,123,130,131,132,133,134,135,136,137]. 

**Figure 9:**
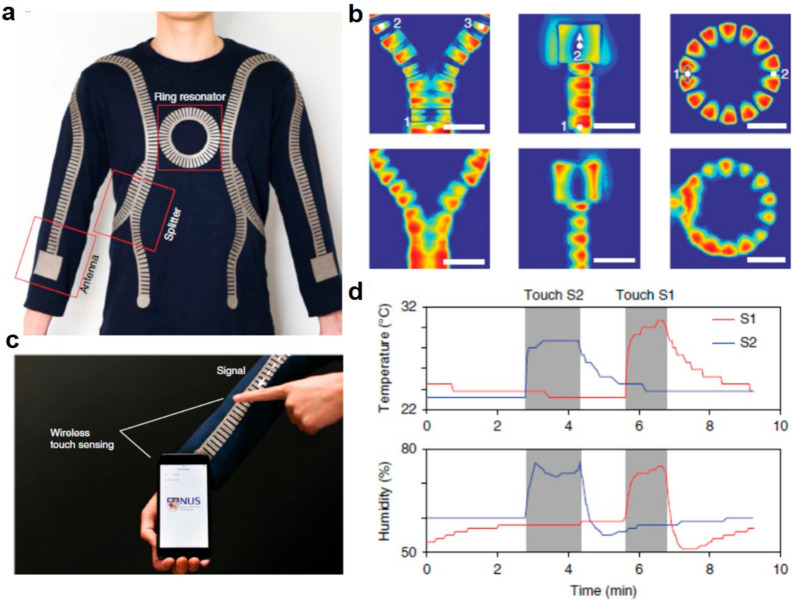
Wearable mechanical metamaterial textile for touching sensing. (**a**) Fabricated metamaterial textile with splitter, antennas and a ring resonator. (**b**) Simulation (top) and experiments (bottom) of the normal component of the electric field. (**c**) Metamaterial textile-based wireless touching sensing and (**d**) detected temperature and humidity signals when touching on shoulder (S1) and on wrist (S2), respectively. Reproduced with permission from [138]. Copyright 2019, Nature.

Previous HMI scenarios are implemented by different kinds of sensors, but integrating these different sensors into a practical network is still challenging, such as the power supply problem and the data collection issue [2,114,131,138]. For convenient wearability, wireless technologies are desired to achieve the body networks, but conventional radio frequency (RF) signals may be transmitted into the ambient space, leading to the low efficiency and useless power consumption. In contrast, the EM waves can be transmitted along the metallic surfaces as surface plasmons, whose evanescent characteristics in the ambient space help to enhance the efficiency of sensing signal transmission. To tune the radio surface plasmon as sensing signal transmission channel, Tian et al. designed a metamaterial textile-based wireless body sensor network for HMI applications, whose transmission efficiency was enhanced by three orders of magnitude compared to the conventional radiative network [138]. The fabricated metamaterial textile is shown in Figure 9a, and there are three basic building blocks on a conventional shirt, namely power divider, antenna, and ring resonator. The simulations and measurements of these three blocks in Figure 9b agree well, demonstrating the different functional roles in the metamaterial textile such as signal distribution and combination, signal radiation, signal filtering, mechanical strain sensing, HMI, etc. To demonstrate the potential for HMI, they mounted a Bluetooth module onto the metamaterial textile to transmit the wireless signals to a smartphone near the body within 10 cm in Figure 9c. When touching, the temperature and humidity signals were detected and displayed on the smartphone robustly in Figure 9d. The metamaterial textile can support robust wireless signal propagation through the metamaterial effect and can be used for multiple HMI applications like gesture sensing, proximity detection, and healthcare monitoring. 

## 5. Conclusions and Perspectives

In summary, we present an overview of the state-of-the-art research progress in metamaterials-enabled sensing for HMI applications. Beginning with the introduction of the various metamaterials-based sensing principles, we first presented the introduction of the metamaterial-based devices from four aspects, i.e., optical sensing, thermal sensing, acoustic sensing, and mechanical sensing. These four aspects are the most common ways for practical HMI scenarios. Then the typical metamaterials-based HMI applications are followed, including the optical metamaterials for human detection, thermal metamaterials for human recognition, acoustic metamaterials for sound recognition, wearable mechanical metamaterials for touching sensing. Overall, all these significant progresses on metamaterials-enabled sensing have advanced the concept of HMI significantly. An overview of such progress in this paper will help us to get the whole picture of the status, to understand the mechanisms, and to stimulate the further exploring. Although metamaterial-enabled sensing for HMI applications have not been investigated intensively and extensively, it is expected that metamaterials in multiple disciplines will benefit for sensing devices for HMI with more compact size, higher accuracy, easier assembly and scalable manufacturing. Here we, as far as we are concerned, discuss and outline several future directions of metamaterial-based sensing for both fundamental research and potential applications in the field of HMI and subfield therein:

- *Flexible metamaterials for sensing*. Most sensing metamaterials or metasurfaces are fabricated on rigid surfaces like the metal/insulator/metal resonator for the easy manipulation of the LSPR effect. However, rigid surfaces are usually not welcome for wearable HMI applications [1,2]. Developing the metamaterial and metasurfaces on flexible and soft substrates are significantly demanded but far from well-developed yet [119,130]. More effort is needed for exploring proper flexible materials and structures for future applications.
- *Integration of detecting and sensing systems*. Most metamaterials can sense the ambient by the modulated light-matter interactions on the surfaces, but these interactions are detected by external complicated systems [63,66,68,139]. Although the sensitivity is improved by the metamaterials, the complicated detecting systems may disable the wearable HMI applications. Further exploring the integration of the detecting and sensing systems is thus important for promoting the metamaterials-based sensing for HMI applications.
- *Metamaterials-enabled multimodal HMI*. For HMI applications, many new technologies emerge in recent decades like VR, AR, MR, IoT, and so on [140,141]. In these technologies, more accurate sensors are significantly needed, but metamaterials-based sensors have not been involved in these HMI scenarios. Combining these new types of HMI with metamaterials is quite interesting and worth of exploring in the future.

## Data Availability

Data may be asked upon reasonable request from the corresponding author.
